# Design, development and production of nano-particle-based anticancer vaccine targeting human aspartyl (asparaginyl) β-hydroxylase (HAAH)

**DOI:** 10.1186/2051-1426-1-S1-P201

**Published:** 2013-11-07

**Authors:** Biswajit Biswas, Solomon Stewart, Kanam Malhotra, Michael Lebowitz, Steven Fuller, Carl Merril, Hossein Ghanbari

**Affiliations:** 1 Panacea Pharmaceuticals Inc., Gaithersburg, MD, USA

## 

HAAH is a protein that is over-expressed in cancer cells and is presented on the surface of the cells and in the growth zone of the tumors. It plays a central role in the etiology of cancer, namely, in proliferation, motility and invasion. It is, however, an embryonic protein important during development and, as such, is a self protein to which the immune system is tolerized. We are targeting HAAH to develop an immunotherapy for cancer. To overcome the self-antigen tolerance of the molecule, we have designed a vaccine entity that contains an immunostimulant and presents HAAH in a manner that is unfamiliar to the body. We have expressed three portions of the HAAH protein, sequences from the N-terminus, middle portion and C-terminus, as fusion proteins (with the gpD bacteriophage antigen) on the surface of bacteriophage lambda, generating 200-300 copies per phage. These vaccine entities have been characterized and are readily and routinely produced at a level of 1012 plaque-forming units (pfu) per liter of E. coli culture. The bacteriophage vaccines have been successfully isolated and purified using tangential flow filtration, a highly scalable process, as well as by PEG precipitation followed by exhaustive dialysis. Both of these processes have reduced bacterial endotoxins to levels within FDA guidelines for formulated human doses. The ease and yield of the manufacturing process allow production of approximately 1000 human doses (based on our anticipated dose requirement for immunogenicity in human subjects) per liter of culture. The bacteriophage can be rendered noninfectious by ultraviolet irradiation; hence referred to as nano-particle-based vaccine. We have demonstrated in previous passive immunotherapy studies that monoclonal antibodies to HAAH inhibit in vitro tumor cell growth, motility and invasiveness and inhibit in vivo tumor growth in mouse xenograft models of cancer. Building on these results, we are using the therapeutic cancer vaccine described here to overcome the self-tolerance to HAAH by the presentation of the altered HAAH antigen (the fusion proteins using HAAH fragments) on a solid phase and the immunomodulatory effect of the bacteriophage itself in recruiting dendritic cells to this antigen. This vaccine should therefore produce a strong polyclonal antibody response and a cellular immune response that will result in enhanced inhibition of tumor cell function and efficacy as an anti-cancer immunotherapeutic agent.

